# Supplementary material for: Patterns of Intron Gain and Loss in Fungi
Source: PLoS Biol. 2004 Nov 30;2(12):e422. doi: 10.1371/journal.pbio.0020422 (PMC532390; doi:10.1371/journal.pbio.0020422)
Supplement: Table S1 — Also available at http://genes.mit.edu/NielsenEtAl/. (4.3 MB ZIP). [file pbio.0020422.st001.zip › NielsenEtAl/html/676.html]

AN5022.1.NCU07196.1.MG08423.1.FG10315.1


```
 CLUSTAL W (1.82) Multiple Sequence Alignments - Introns Inserted


Sequence 1: NCU07196.1	197 aa
Sequence 2: FG10315.1	196 aa
Sequence 3: MG08423.1	196 aa
Sequence 4: AN5022.1	202 aa
Alignment Length: 211 aa
Number Identitical Residues: 75 aa
Alignment Score (without introns) 4560


MG08423.1 	MSRRPPRGEYIET0DTGNKVSRKAVLVGTQNIMLGGKTVIQPEVMIRGDLTRTSTS----
NCU07196.1	MSRRPPRGEYIET~DTGNKVARKATLVGTQNIMLGGKTVIQPDVMIRGDLARTIPP----
FG10315.1 	MSRRPPKGEYIET0DTGNKVARKAVLVGTQNIMLGGKTVIQPEVMIRGDLARTVTA----
AN5022.1  	MPPKAPKGEYIET0ETGNKVSRRSQIHGTHHISLGGKSIIMADAVVRGDLFRSSSSQSQS
          	*. :.*:****** :*****:*:: : **::* ****::* .:.::**** *: ...:.:

MG08423.1 	-----GSSSS--AESKTAVAIGRYCFLSRGSCLKPPSRLHKG2TFTHIPLRIGDHVFVGE
NCU07196.1	-----AQSASGGPANNTAVAIGRYCFLSRGCCLRPPGRMYKG2VFTFMPLRLGDHVFVGP
FG10315.1 	-----SSSGS-APANNTAVAIGRYCFLARGVLLRPPGRMYKG~AFTYMPLRIGDHVFVGQ
AN5022.1  	QSQSQSQSGSGAGNNNIAISIGRYTFISRSAILRPPSRLSRG~VHTYTTLHIGSHVFVGE
          	.:.:...*.*..  .: *::**** *::*.  *:**.*: :* ..*. .*::*.***** 

MG08423.1 	RTVVSAASVGSYVHIGHDAVVGDLVVIKDYVRVLDGAVIAPNTVVPSFSIVAGRPARVIG
NCU07196.1	GTVVQAAQIGNHVHIGGKVVIGEFAIIKDYVKVLDGSVVPPNMVIPSFSIVAGQPARIIG
FG10315.1 	GTVVQAAAVGNHVQIGRDCTIGEFAILKDYVQVLDGSVVPPNMVIPSFSIVAGQPAKVIG
AN5022.1  	RSIVEAAKVEDNVTIGKDCVIGSMAILKERCQVLDGCVVPGGMVVPSHCVVGGQPARIVG
          	 ::*.** : . * ** . .:*.:.::*:  :****.*:. . *:**..:*.*:**:::*

MG08423.1 	EVPEG-GHEAFQ---LRELYKTVGNNPQPAAVV
NCU07196.1	EIPEG-GIEAFE---LRDLYKTVGNNPQPTAP-
FG10315.1 	EIPEG-GHEEFE---LRDMYKTVGNNPQPPAS-
AN5022.1  	DTPIAYGVEGLEGGLSRERYRSIR---------
          	: * . * * ::..  *: *:::
```
